# A novel LC-MS/MS analysis of vitamin D metabolites in mice serum and hair: impact of diet and light exposure

**DOI:** 10.3389/fendo.2025.1494393

**Published:** 2025-02-05

**Authors:** Muhammad K. Hakeem, Asma Al-Menhali, Sampath K. Elangovan, Iltaf Shah

**Affiliations:** ^1^ Department of Chemistry, College of Science, United Arab Emirates University (UAEU), Al Ain, United Arab Emirates; ^2^ Department of Biology, College of Sciences, United Arab Emirates University (UAEU), Al Ain, United Arab Emirates

**Keywords:** LC-MS, vitamin D metabolites, mice serum, mice hair, 25OHD 25-hydroxyvitamin D

## Abstract

**Introduction:**

Numerous physiological systems, such as the functioning of the immune system, bone health, and the regulation of expression of genes, depend critically on vitamin D. Considering the significance of vitamin D for health, it is critical to understand how it is metabolized and the factors that affect its levels.

**Methods:**

The objective of this study was to develop and validate an LC-MS/MS method to examine the effects of light exposure and dietary vitamin D consumption on the levels of vitamin D and its metabolites in a mouse model under consistent growth conditions throughout the year. Serum and hair samples from mice were analyzed under various experimental conditions for vitamin D and its metabolites using liquid chromatography-tandem mass spectrometry (LC-MS/MS). The experimental conditions included a vitamin D-deficient diet, a vitamin D-standard diet, and changes in ambient light exposure ranging from complete darkness to a regular light-dark cycle.

**Results:**

Mice fed a standard vitamin D diet and exposed to a regular light-dark cycle exhibited significantly higher levels of 25OHD_3_ in both serum and hair, indicating the synergistic effect of dietary vitamin D intake and light exposure. Mice fed a standard vitamin D diet but kept in continuous darkness showed moderately elevated 25OHD_3_ levels, demonstrating the efficacy of dietary vitamin D in maintaining adequate levels despite the absence of light. Conversely, mice fed a vitamin D-deficient diet and housed in darkness displayed 25OHD_3_ levels below the limit of quantification, highlighting the combined detrimental effects of dietary deficiency and lack of light exposure.

**Discussion:**

This study provides valuable insights into the complex interplay between dietary vitamin D intake, light exposure, and the regulation of vitamin D metabolism in mice. Moreover, our results underscore the potential implications for human health, suggesting the importance of adequate vitamin D intake and sunlight exposure in maintaining optimal vitamin D levels. Further research in this area has the potential to unveil additional factors influencing vitamin D metabolism, offering valuable insights into strategies for optimizing vitamin D levels in both animal models and human subjects.

## 1 Introduction

Vitamin D is a vital, fat-soluble nutrient which plays a pivotal part in maintaining calcium and phosphate balance within the body (1, 2). This balance of calcium and phosphate is crucial for healthy bone development and mineralization (3, 4). The calcium and phosphorus balance also influence the conditions such as hypertension, chronic kidney disease, psoriasis, cancer, and several autoimmune diseases (5–9). Beyond its primary functions, it significantly influences various physiological processes, including the immune system’s regulation, cellular growth, and differentiation (10–13). In addition to its nutritional role, vitamin D acts as a steroid hormone integral to numerous biological functions across the cardiovascular, nervous, skeletal, and reproductive systems (14–16).

Vitamin D exists in two primary forms within the human body: vitamin D_2_ (ergocalciferol) and vitamin D_3_ (cholecalciferol), with D_3_ recognized for its paramount biological importance (17, 18). After being exposed to ultraviolet B radiation (UVB) from the sun, 7-dehydrocholesterol is converted in the skin to vitamin D_3_ (19). Vitamin D_3_ is also obtained from dietary sources. Vitamin D_3_ requires activation through two hydroxylation steps (20). It is first converted to 25-hydroxyvitamin D_3_ (25OHD_3_) in the liver (21), and then to 1α25(OH)_2_D_3_, the active form, in the kidneys (22). The regulation of this pathway is tightly controlled by feedback mechanisms involving calcium and phosphate homeostasis, as well as parathyroid hormone (PTH) levels. Dysregulation of these processes can lead to endocrine disorders such as rickets, osteomalacia, and vitamin D-dependent hypercalcemia (23–27). Our study’s analytical method enables precise quantification of vitamin D metabolites, including epimers, which are emerging as critical indicators of endocrine health. This capability offers insights into vitamin D metabolism in physiological and pathological states, advancing our understanding of endocrine dysfunctions.

Unlike D_3_, vitamin D_2_ is exclusively obtained through the diet and metabolized similarly to yield its active forms and respective epimers (28, 29). The most potent form, 1α25(OH)_2_D_3_, exerts its effects through genomic and non-genomic pathways, largely due to its affinity for vitamin D receptors (VDRs), including both the nuclear and membrane-bound receptors (22, 30). Interestingly, vitamin D epimers also contribute to physiological health, affecting bone mineral density and growth (31, 32). Notably, these epimers have been observed at elevated levels in mice receiving dietary vitamin D_3_ supplementation (29).

In humans, newborns and children exhibit elevated concentrations of vitamin D epimers reaching up to 60% till reaching the age of one year (33). There is a range of 0 to 45% for the ratio of 3-epi-25OHD to 25OHD_3_. Notably, the concentration of 3-epi-25OHD_3_ increases linearly with vitamin D_3_ medication (29, 34). According to a research, certain pregnant women and babies have higher than average concentrations of 3-epi-25OHD_3_, and the reason for these higher-than-average concentrations throughout infancy is probably early embryogenesis (31, 35).

Vitamin D’s role as an endocrine regulator extends beyond calcium and phosphate homeostasis. Its active form, 1α25(OH)_23_, binds to VDRs to modulate the transcription of genes involved in bone metabolism, immune responses, and cell proliferation (11, 36, 37). Non-genomic actions, mediated by membrane-bound VDRs, rapidly activate signaling cascades such as MAPK and PI3K/AKT, influencing cellular responses (38, 39). Dysregulation in these pathways contributes to endocrine disorders such as rickets, osteoporosis, and autoimmune diseases, underlining the clinical importance of accurately quantifying vitamin D and its metabolites (40–42).

Given how important vitamin D is to the body, several analytical techniques have been developed for investigating the metabolites of vitamin D in samples from both humans and animals. The low concentration of vitamin D and metabolites in biological matrices, together with the availability of metabolites that are structurally identical, provide significant analytical problems for this research (43–45). The most effective of these approaches is the LC-MS/MS method, overcoming the limitations associated with immunoassay techniques while offering high sensitivity and specificity (46). Its short run time, ability to separate interfering co-eluting epimers and isobar effectively, and use of an internal standard that allows for the evaluation of instrumental and matrix-related effects are among its few noteworthy benefits (47–51).

Animal models, particularly rodents, have been instrumental in elucidating the metabolic pathways of vitamin D and the physiological consequences of its deficiency or excess (52, 53). However, fewer studies have comprehensively analyzed the effects of dietary variation and environmental factors, such as light exposure, on vitamin D metabolism in these models (54, 55).In addition to serum, hair has emerged as a valuable matrix for vitamin D metabolite analysis due to its unique properties. Hair offers several advantages as a non-invasive, integrative biomarker that reflects long-term exposure to nutrients and environmental factors, making it suitable for assessing vitamin D accumulation over extended periods. Unlike serum, which provides a snapshot of current vitamin D status, hair accumulates metabolites over time, allowing for retrospective evaluation of vitamin D levels. Studies suggest that vitamin D and its metabolites are deposited in hair, providing a more stable record of nutritional status that is less affected by short-term fluctuations. Thus, analyzing vitamin D in hair complements serum analysis and provides a holistic view of vitamin D dynamics in response to dietary and environmental changes (49, 56–58).

In this study, our primary objective was to enhance and validate an LC-MS/MS methodology for the comprehensive analysis of vitamin D and its metabolites in both mouse hair and serum samples. Additionally, our study aimed to elucidate the impact of varying vitamin D dietary routines and exposure to light on the concentrations of vitamin D and its metabolites within the mouse population. The study by Zgaga et al. (59) was the first to extract and quantify 25OHD_3_ from human hair, highlighting hair’s potential for tracking vitamin D levels. Our research extends this work by focusing on mouse hair and serum under varied growth conditions. Notably, external factors such as diet and light exposure may influence vitamin D levels differently across species, emphasizing the need to understand these distinctions. This is essential for translating findings from animal models to potential human applications. To the best of our knowledge, no prior study has developed a method for the quantification of vitamin D in mouse hair and effect of different growth conditions highlighting the innovative aspect of our approach. Building upon our prior investigations into vitamin D analysis in mice serum (29), we have expanded our research to incorporate mice hair sample analysis and to investigate the effect of different growth and diet conditions. This comprehensive study aims to elucidate the relationship between vitamin D and its metabolites concentration in mice serum and hair and to examine the impact of different diet and light exposure conditions on these analytes, providing valuable insights into the dynamics of vitamin D metabolism in mouse population.

## 2 Materials and methods

### 2.1 Standards and reagents

A range of standards and reagents is employed in this study to ensure accurate and precise analysis. Vitamin D_3_, vitamin D_2_, 25OHD_3_, 25OHD_2_, 3-epi-25OHD_3_, 3-epi-25OHD_2_, 1α25(OH)_2_D_2_, and 1α25(OH)_2_D_3_, 7αC4 and Internal standard (ISTD) [25-hydroxyvitamin-D_3_ (6,19,19-d_3_)] were utilized as standards in our experiment. LC-MS grade Water, methanol, acetonitrile, ethyl acetate, formic acid, ammonium formate, and ammonium hydroxide were used for the preparation of the solutions during the study. LABCO LLC in Dubai, UAE, supplied all of these chemicals, which were purchased from Sigma Aldrich. Isopropanol, hexane, and dichloromethane sourced from Emirates Scientific & Technical Supplies LLC in Dubai, UAE, were also integral components of our experimental setup. These meticulously selected standards and reagents were crucial for achieving reliable and reproducible results throughout our study.

### 2.2 Growth condition

**Figure 1:**
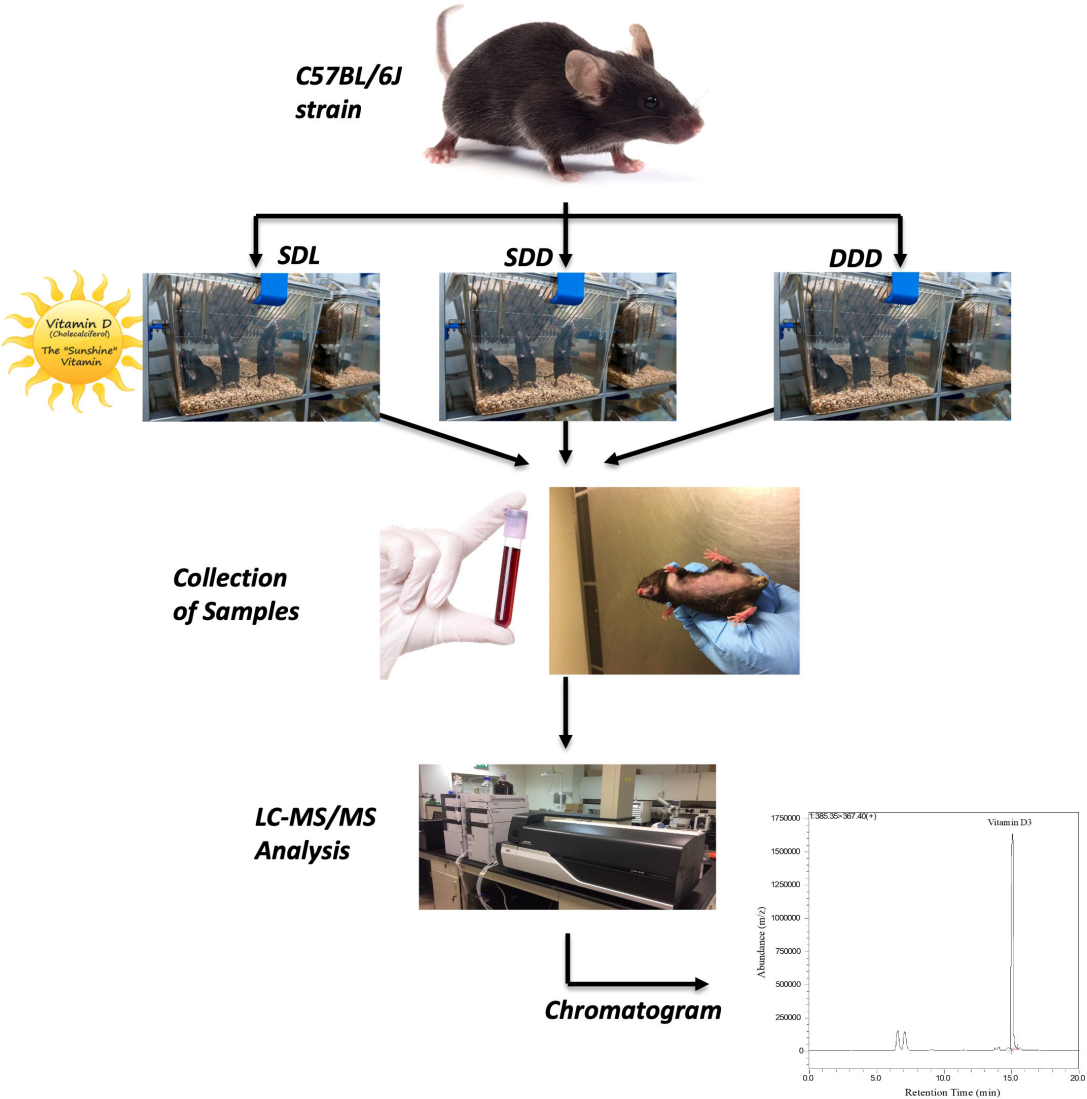
Schematic representation of the experimental workflow.

The Ethics Committee of United Arab Emirates University (UAEU) granted approval for the study (Ref # ERA_2017_5684). The mice used in this study were of the C57BL/6J strain, which is widely recognized for its genetic predisposition to lower bone density. This strain’s skeletal phenotype is well-documented, characterized by reduced bone formation and altered bone turnover dynamics (60, 61). Mice were kept in the animal facility of the UAEU Faculty of Medicine and Health Sciences in a specialized pathogen-free (SPF) environment. Upon weaning at 3 weeks old, a cohort of 40 mice was carefully allocated to three distinct groups, each characterized by unique dietary compositions and light exposure routines. The first group, designated as SDL, consisted of 15 mice and received a standard-vitamin D diet while being exposed to alternating 12-hour cycles of UV light and darkness. The second group, labelled as SDD (control group), also included 15 mice and was provided with a standard-vitamin D diet (Standard AIN-93G Rodent Diet with 1000 IU VD3, D10012Gi, Research Diet) but was housed in continuous darkness. Lastly, the third experimental group, DDD, comprised 10 mice and was fed a diet deficient in vitamin D (AIN-93G Growing Rodent Diet with 25 IU VD3/kg of diet, D17053003i, Research Diet), also maintained in darkness. Each group was housed in separate cages to ensure consistency in exposure conditions. Mice were housed in temperature-controlled rooms maintained at 22 ± 2°C, with humidity levels of 50 ± 5%. For groups exposed to UV light, the wavelength was set at 280–320 nm, which effectively supports the cutaneous synthesis of vitamin D_3_. Cages were kept separate to ensure controlled light exposure and dietary conditions. These conditions were rigorously maintained over a period of 12 months. A 12-month study duration was chosen to capture long-term endocrine adaptations, as vitamin D metabolism and deposition, particularly in hair, are influenced by cumulative dietary and environmental exposures. This duration aligns with previous research indicating that hormonal adaptations in rodents occur over extended periods, reflecting chronic physiological changes. Sample sizes were determined based on power analyses, ensuring sufficient statistical power (>80%) to detect significant differences in vitamin D metabolite concentrations between groups and time points. **Figure 1** below provides a schematic representation of the experimental setup, including the C57BL/6J mice strain, dietary and light exposure conditions, sample collection, and subsequent LC-MS/MS analysis.

### 2.3 Anesthesia protocol

In our study, the mice were anesthetized using diethyl ether at a concentration of 1.9%, which was vaporized in a sealed chamber. Specifically, 80 microliters of diethyl ether per liter of container volume were used to create the anesthetic atmosphere. The mice were placed in the chamber, and the induction of anesthesia was monitored by observing the loss of the righting reflex and the absence of response to tactile stimuli, indicating adequate anesthesia. The duration of exposure was carefully controlled to ensure humane treatment and avoid prolonged exposure. All procedures were performed in compliance with ethical guidelines and institutional protocols to ensure the welfare of the animals (62, 63).

### 2.4 Hair sample collection & pre-treatment

Hair samples were collected to analyze vitamin D metabolite deposition. To minimize variability arising from the hair growth cycle, the growth cycle was synchronized by shaving hair at the onset of the study. Samples were collected after six months (6-M) and 12 months (12-M) of regrowth, ensuring consistency across the experimental groups. Hair samples were consistently obtained from the dorsal region of the mice due to its uniform growth characteristics and reduced susceptibility to external contaminants. Each hair sample was carefully shaved, weighed, and placed in labelled plastic bags, maintaining clear identification and stored at 4°C to prevent degradation. In preparation for analysis, collected hair samples corresponding were subjected to pre-treatment procedures. This involved washing the hair samples with a 1:1 methanol/water mixture to remove sweat, sebum, and contaminants. The washed hair samples were then dried and pulverized into a fine powder using a mini ball mill. A precise amount of 30 mg of grounded hair was weighed and transferred to labelled borosilicate culture tubes for analysis.

Additionally, blank hair samples were included in the analysis to serve as controls. These blank hair samples were obtained from the DDD group of mice, which were maintained on a vitamin D-deficient diet in dark. These samples underwent a rigorous selection process to ensure their cleanliness and absence of interfering substances. Selected hair samples were subjected to an LC-MS/MS analysis for this purpose, and no interference peaks were seen during the retention time (RT) of any vitamin D metabolites. After selection, the calibration curves and quality controls were prepared using one of the cleanest blank samples.

### 2.5 Hair sample extraction

For the extraction of vitamin D and its metabolites from the hair samples, a systematic approach was followed. Standards and the internal standard [25-hydroxyvitamin-D_3_ (6,19,19-d_3_)] were introduced to the hair samples that were being investigated, calibrants, and quality controls. After they had been sonicated in a 50:50 v/v combination of methanol and water, the top layer of these samples was collected by centrifugation at 4,000 rpm for 10 minutes. The obtained layer was dried and filtered using nitrogen gas before being reconstituted in a solution of methanol and water (50:50 v/v). subsequently, the LCMS/MS system was equipped with the reconstituted samples to begin the analysis. The blank samples were prepared as per the given extraction procedure without the addition of analyte. These samples were confirmed to contain no interference peaks at the RT of analyte, ensuring their suitability as blanks for the analysis.

### 2.6 Serum sample collection and pre-treatment

To investigate the presence of vitamin D and its metabolites in serum, blood samples were taken from each of the three experimental mouse groups (DDD, SDD, and SDL) at two time points: 6 months and 12 months. To reduce circadian variability, blood collection was performed consistently in the morning, between 8:00 and 10:00 AM. Each mouse was carefully anesthetized to minimize stress and discomfort. Approximately 300 µL of blood was drawn from each mouse using BD vacutainers (part no. 367957) to ensure uniformity and reliability of the samples. The blood samples were collected, centrifuged at 1300 rpm for 15 minutes at 4°C, and then kept at -80°C. Stability tests confirmed that vitamin D metabolites remained stable for up to 12 months under these storage conditions, with less than 10% variation in measured concentrations. During thawing and preparation, light exposure was minimized to prevent degradation. Upon collection, the mouse serum samples underwent a series of preparatory steps to ensure optimal conditions for subsequent analysis. Each serum sample (0.100 mL) was allowed to thaw at room temperature for 15 minutes to prevent thermal shock and maintain sample integrity. After thawing, the samples were vortexed thoroughly to ensure homogeneity and minimize any potential for sample stratification. Except for the blank, 50 µL of a working internal standard solution containing 25-hydroxyvitamin-D_3_-(6,19,19-d_3_) was carefully added to all calibration standards, quality controls, and serum samples in order to aid accurate measurement. To ensure precise calibration of the analytical instrument and to simulate the matrix effect, calibration standards and quality controls samples were carefully prepared in blank serum.

### 2.7 Extraction of serum sample

Following pre-treatment, the serum samples underwent extraction to isolate vitamin D and its metabolites for subsequent analysis. Liquid-liquid extraction was performed using a well-established protocol with a mixture of hexane: ethyl acetate (9:1; v/v). This extraction method ensured efficient extraction of all analytes from the serum matrix while minimizing interference from other components. The extracted mixture was vigorously vortexed for 10 minutes to ensure thorough mixing of the solvent and sample components. Subsequently, the mixture underwent centrifugation at 1320 rpm for 10 minutes to separate the organic layer containing the analytes of interest from the aqueous layer. Pasteur pipettes were used to gently decant the translucent organic layer of the supernatant after centrifugation and transport it to other test tubes. To maximize extraction efficiency, the remaining lower layer underwent three additional extraction cycles using the same protocol. All extracted samples were then pooled together and dried under N_2_ gas in a sample concentrator to remove residual solvent. The dried residue was reconstituted in a carefully prepared mixture of 100 µL LC-MS/MS-grade methanol and water (75:25, v/v) to ensure optimal conditions for subsequent analysis.

### 2.8 LC-MS/MS system

The analytical system employed for this study included a tandem mass spectrometer, the model 8060 from Shimadzu, Japan, integrated with a Nexera ultra-high-performance liquid chromatography (UHPLC) setup, Nexera X2 series, also from Shimadzu, Japan. The UHPLC system was equipped with essential components including a pump, an auto-sampler, a column oven, and a degasser, enhancing the sensitivity of our analysis using columns that were both narrow bore and packed with small particles, capable of withstanding high pressures. For ionization, the mass spectrometer operated in the positive electrospray ionization (ESI) mode. Data acquisition and analysis were conducted using Shimadzu’s Lab-Solutions software, ensuring precise data handling. We optimized the mass spectrometer’s operational settings such as nebulizer, drying and heating gas flows, interface, and block temperatures to maximize the detection and quantification accuracy for vitamin D metabolites. Specific conditions were set as follows: nebulizing gas at 2 L/min, drying gas at 8 L/min, and heating gas also at 8 L/min, with interface and block temperatures maintained at 300°C and 400°C, respectively.

To prevent any degradation of samples by light exposure, sample handling and LC-MS/MS injections were performed under ambient lighting conditions. Analyte stability was assessed under various conditions, including room temperature, freeze-thaw cycles, and prolonged storage at -80°C. All metabolites demonstrated stability, with less than 10% variation in measured concentrations. Metabolite separation was achieved using an Ascentis Express F5 column, measuring 150 mm by 2.1 mm and with a 2.7 µm particle size, maintained at 40°C complemented by a pre-column guard. The injection volume for all samples was set at 10 μL. A methanol-water solution (50:50, v/v) was employed to rinse the injector needle between runs, mitigating carryover and contamination risks. The mobile phase consisted of 5 mM ammonium formate in water (Phase A) and methanol (Phase B), each containing 5 mM ammonium formate, propelled at a flow rate of 0.5 mL/min via a binary gradient pump system. The gradient program was meticulously set to begin with 25% Phase A and 75% Phase B for the first 11 minutes, transitioning to 100% Phase B from 11 to 15 minutes, maintained at this composition till the 16th minute, and then returning to the initial conditions of 25% A and 75% B from 16.1 to 20 minutes to ensure optimal separation.

In the analysis of vitamin D and its metabolites, an electrospray ionization (ESI) source served as the primary method for ionization and protonation, yielding molecular ions of the form [M + H]^+^. To ensure maximum sensitivity and accuracy in quantification, optimization of multiple reaction monitoring (MRM) parameters was conducted. The optimization protocol aimed to identify the most sensitive MRM transition for each analyte, focusing on precursor and product ions that exhibited the highest sensitivity. Our method involved the use of three MRM transitions for each analyte, comprising one quantifier ion and two qualifier ions. To ensure optimal data acquisition, we set the target cycle time at 0.5 seconds, allowing for efficient sampling while maintaining sensitivity. Additionally, a retention-time window of 90 seconds and an interscan delay of 3 milliseconds were implemented to accurately capture the analytes of interest.

Through rigorous experimentation and data analysis, the most sensitive precursor and product ions were selected for LC-MS/MS analysis, enhancing the detection capabilities of the instrument. Interestingly, chromatographic separation facilitated by the LC system allowed for clear differentiation between closely related compounds such as 25OHD_3_, 3-epi-25OHD_3_, and 7αC4, despite their identical ionization precursor and product ions, as well as collision energies. Similarly, despite having the identical precursor, product, and collision energies, differentiation between 25OHD_2_ and 3-epi-25OHD_2_ was accomplished based on retention times. **Table 1** provides a detailed presentation of the chosen MRM transitions and the associated parameters, such as precursor/product ions, retention times, and collision energy.

**Table 1:** MRM parameters for vitamin D and its metabolites.

No.	Analytes	Retention Time (min)	Precursor Q1 (m/z)	Product Q3 (m/z)	Collision Energy (eV)
1	Vitamin D_3_	15.212	385.0	367.0	–13
				259.0	–16
				91.0	–56
2	Vitamin D_2_	15.180	397.1	379.4	–17
				69.0	–19
3	25OHD_3_	6.991	383.2	365.3	–15
				107.1	–30
4	25OHD_2_	7.809	395.1	377.3	–17
				81.1	–38
5	3-epi-25OHD_3_	7.701	383.2	365.3	–15
				107.1	–30
6	3-epi-25OHD_2_	8.401	395.1	377.1	–17
				81.1	–38
7	1α25(OH)_2_D_3_	3.799	399.1	381.3	–14
8	1α25(OH)_2_D_2_	3.989	411.1	135.3	–13
				133.1	–12
9	7αC4	14.501	401.5	383.25	-16
				97.1	-29
				91.2	-23
10	ISTD [25 hydroxyvitamin-D_3_(6,19,19-d_3_)]	7.010	386.3	368.2	–15
				257.2	–183
				95.2	–35

The validation process for the current study followed strict guidelines set forth by the US Food and Drug Admin.

### 2.9 Method validation

The validation process for the current study followed strict guidelines set forth by the US Food and Drug Administration (FDA) to ensure the reliability and accuracy of the analytical method (64). Optimized Multiple Reaction Monitoring (MRM) parameters were precisely established for the analysis of vitamin D and its metabolites using LC-MS/MS. The MRM mode enabled the tracking of protonated molecules ([M+H]^+^), precursor ions, and diagnostic product ions, ensuring specificity and sensitivity in compound detection. Chromatographic separation allowed differentiation of closely related compounds, such as 25OHD_3_ and 3-epi-25OHD_3_, based on their retention times. Given the presence of other steroid hormones in serum and hair samples, matrix effects were evaluated using post-extraction spiked samples. Ion suppression/enhancement was quantified by comparing responses from spiked extracts to those from neat solutions. No significant interference was observed for the quantification of vitamin D metabolites. To further enhance the rigor of the validation process, stability studies were conducted to evaluate the resilience of vitamin D metabolites under physiological conditions. Stability was assessed under ambient lighting, at room temperature for up to 24 hours, across three freeze-thaw cycles, and during long-term storage at -80°C. The metabolites demonstrated excellent stability, with less than 10% variation in concentrations under all tested conditions, ensuring reliable quantification. Calibration curves were prepared in blank matrices to simulate the physiological environment, minimizing the impact of other hormones.

To evaluate linearity, specificity, and accuracy, a series of quality control samples quality control low (QCL), quality control medium (QCM), and quality control high (QCH) were prepared at different concentrations covering the entire analytical range. These samples were subjected to LC-MS/MS analysis alongside calibration curves to assess the method’s ability to accurately quantify vitamin D metabolites. Analyzing the signal-to-noise (S/N) ratio and comparing it to the lowest concentration of the analyte allowed for the determination of the LOD. To do this, the analyte concentration had to be gradually lowered until a response three times the background level was seen. The blank serum was used as the matrix for the calibration curve and quality controls (QCs), which were made by combining albumin serum with phosphate-buffered saline. The linearity of the calibration curves was assessed using regression analysis, ensuring that the method provided accurate measurements across the specified concentration range. By computing the % CV and % Accuracy based on the examination of quality control samples, precision and accuracy were evaluated. % CV stands for coefficient of variation, and it was computed as $\frac{Standard\;Deviation}{Mean\;}\times 100$ . The quality control samples’ % accuracy was determined by taking the $\left[\frac{Mean\;value}{Nominal\;value}\times 100\right]$ formula. These parameters provided insights into the method’s reproducibility and reliability in quantifying vitamin D metabolites in mice serum and hair samples. Recovery experiments were conducted to evaluate the method’s efficiency in extracting vitamin D metabolites from serum and hair samples. Serum and hair samples were spiked with known quantities of vitamin D metabolites, followed by extraction and the validated LC-MS/MS technique was then used for analysis. The formula for calculating the absolute percentage recovery is $\left[\frac{Mean\;unextracted\;QC\;value}{Mean\;extracted\;QC\;value}\times 100\right]$ , yielding a measure of the method’s extraction efficiency and accuracy.

The serum and tissue samples used in this study were part of a broader research project that investigated multiple aspects of vitamin D metabolism, including its interactions with dietary and environmental factors, as detailed in our prior publications (65–67). These studies focused on the interplay between vitamin D metabolism, gut microbiota, and metabolic pathways, utilizing the available biological materials comprehensively. As a result, the remaining samples were fully utilized for complementary analyses, and no further biological materials remain for additional investigations. Consequently, while we acknowledge the importance of measuring parathyroid hormone (PTH), fibroblast growth factor 23 (FGF23), and the expression of enzymes such as CYP27B1 and CYP24A1 to provide a more complete understanding of the vitamin D endocrine axis, it was not feasible to perform these analyses within the scope of this study due to sample unavailability. Despite these limitations, the current work provides valuable insights into the impact of dietary vitamin D and light exposure on the metabolite profile of vitamin D in serum and hair, laying the groundwork for future studies.

### 2.10 Statistical analysis:

A statistical analysis was conducted to compare the levels of vitamin D metabolites across the three experimental groups (SDL, SDD, and DDD) and over the two time points (6 months and 12 months). One-way ANOVA was performed to compare the means of vitamin D metabolites levels between the groups at both time points. The results were considered statistically significant when p < 0.05. *Post-hoc* analysis using Tukey’s HSD test was applied to further assess differences between groups. Additionally, effect size calculations were included using Cohen’s d to assess the magnitude of differences between the SDL and DDD groups, and partial eta-squared values were calculated to determine the strength of the ANOVA effects. Paired t-tests were used to evaluate the change in vitamin D metabolites levels within each group from 6 months to 12 months.

## 3 Results & discussion

### 3.1 Hair validation results

**Figure 2:**
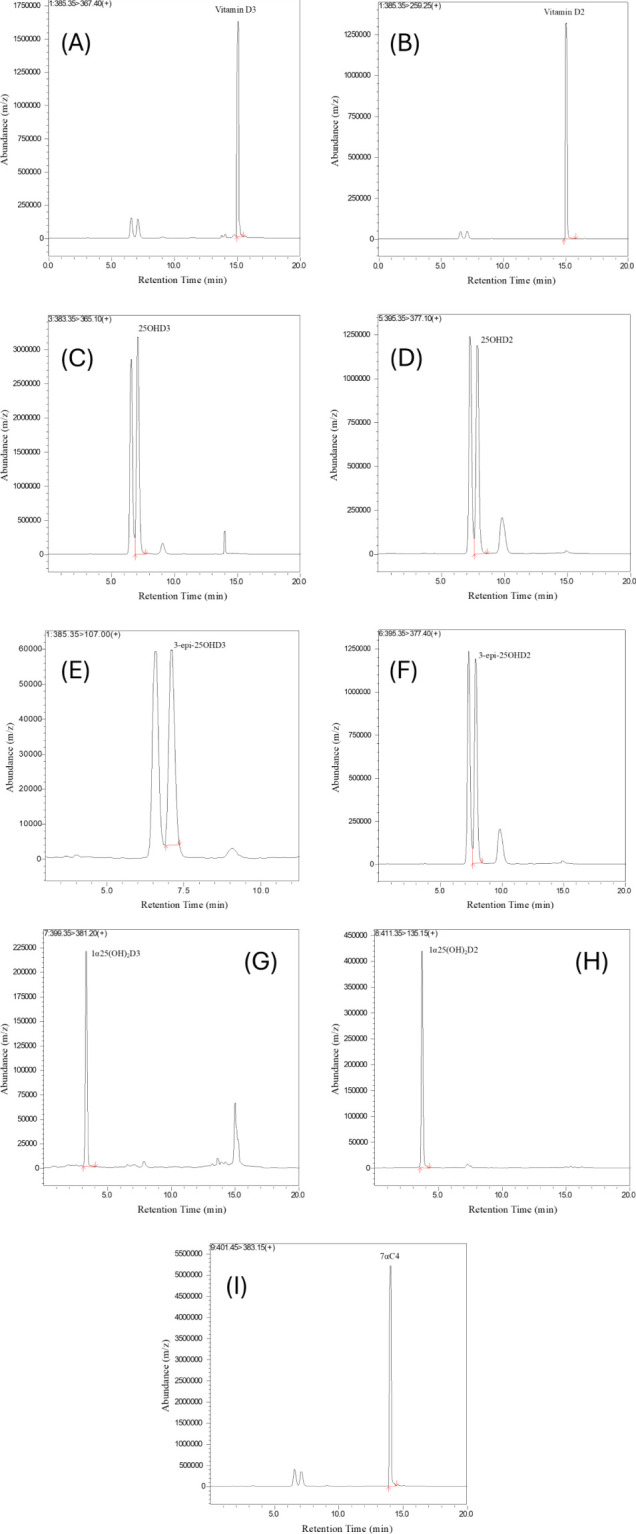
Chromatograms of Internal standard and Vitamin D metabolites **(A)** Vitamin D_3_ **(B)** Vitamin D_2_ **(C)** 25OHD_3_ **(D)** 25OHD_2_ **(E)** 3-epi-25OHD_3_ **(F)** 3-epi-25OHD_2_ **(G)** 1α25(OH)_2_D_3_ **(H)** 1α25(OH)_2_D_2_ **(I)** 7αC4 in spiked hair samples.

Validation of the LC-MS/MS method for analyzing vitamin D and its metabolites in hair samples involved a comprehensive assessment of precision, accuracy, linearity, and recovery values for multiple analytes. LC-MS/MS offered exceptional sensitivity, allowing for precise identification of analytes based on retention time on the LC column and characteristic fragmentation reactions. During the validation process, we carefully examined the retention times and signal intensities of the vitamin D metabolites and internal standard (ISTD). This ensured a strong correlation between the concentration of each metabolite and its signal strength, allowing for accurate quantification across different concentration levels. **Figure 2** provides visual representations of Vitamin D metabolites in spiked hair samples along with ISTD under optimal conditions.

The chromatograms in **Figure 2** were obtained by spiking 240 pg/mg (HQC) of standards in blank hair samples. The chromatograms show well-defined and sharp peaks at their specific retention time for each vitamin D metabolites, highlighting the method’s precision and ability to accurately measure the target compounds. The clear and separate peaks are crucial for accurate quantification, ensuring that each vitamin D metabolite is accurately identified and measured. Additionally, **Figure 2** demonstrates the method’s selectivity by distinguishing target analytes from potential interferences in the sample matrix. These peaks in (**Figures 2A–I**) reinforce the method’s reliability and suitability for precise quantification of vitamin D metabolites in mouse serum and hair samples. These chromatographic results demonstrate the method’s effectiveness thereby enhancing the reliability of the analytical approach for vitamin D analysis.

The method validation encompassed key vitamin D compounds including vitamin D_3_, vitamin D_2_, 25OHD_2_, 25OHD_3_, 1α25(OH)_2_D_2_, 1α25(OH)_2_D_3_, 3-epi-25OHD_3_, and 3-epi-25OHD_2_. Recovery rates, as well as intra- and inter-day precision and accuracy values, were carefully evaluated and presented in a **Table 2**. All analytes had a lower limit of detection (LOD) of 5 pg/mg, and all vitamin D metabolites had a linear range of 5 pg/mg to 300 pg/mg. The FDA guidelines’ criterion for linearity were met when the linear regression (R^2^) values for the calibration curve were equal to or higher than 0.986.

**Table 2:** Method validation results for intraday/interday precision, accuracy, and recovery.

Analytes	QC’s (pg/mg)	Intraday		Interday		% Recovery
		%CV	%Accuracy	%CV	%Accuracy	
Vitamin D_3_	14	14.90	106.1	14.82	109.8	79
	90	7.99	97.4	7.95	99.4	77
	240	6.20	99.9	8.39	100.2	88
Vitamin D_2_	14	13.21	105.1	12.9	103.2	88
	90	9.81	98.5	7.1	99.8	89
	240	5.64	99.4	6.2	101.3	92
25OHD_3_	14	13.22	99.9	13.4	102.1	86
	90	9.90	95.3	8.7	99.8	89
	240	11.00	99.7	9.3	98.7	94
25OHD_2_	14	14.78	107.9	11.2	101.8	82
	90	8.01	99.4	7.3	98.9	90
	240	5.21	99.9	4.3	97.8	80
1α25(OH)_2_D_3_	14	12.81	107.2	14.7	104.5	75
	90	7.21	102.3	10.9	102.6	96
	240	3.20	100.1	8.9	99.9	78
1α25(OH)_2_D_2_	14	13.78	98.9	10.2	101.5	76
	90	5.92	99.6	5.7	93.1	87
	240	2.10	89.6	3.2	91.3	89
3-epi-25OHD_3_	14	12.77	98.3	13.3	106.2	78
	90	13.20	102.6	9.1	95.3	89
	240	14.01	91.9	10.6	108.1	97
3-epi-25OHD_2_	14	13.24	99.3	12.3	101.2	92
	90	5.01	98.7	6.1	97.8	94
	240	7.99	101.2	7.2	96.9	97
7αC4	14	14.36	97.2	12.7	103.2	75
	90	7.22	91.7	5.8	97.1	79
	240	2.78	89.3	3.7	93.5	72

The method’s effectiveness was further demonstrated through the identification of precursor and product ion pairs, alongside optimized collision energies used for the multiple reaction monitoring (MRM) method. The LC retention time and MRM parameters for the analysis of 25OHD_3_ in hair samples were aligned with the validated procedure, underscoring the method’s robustness and reliability.

### 3.2 Hair sample analysis

In this experimental study, we provide the measurement of vitamin D metabolites in the three groups of mice: the group fed a diet deficient in vitamin D and kept in the dark (DDD), the group given a diet rich in vitamin D and kept in the dark (SDD), and the group exposed to light in addition to the regular diet of vitamin D (SDL). We conducted sample collection and analysis at two intervals, specifically at the 6^th^ month and 12^th^ month marks, spanning over the course of a year. Our objective was to assess the impact of both diet and exposure of radiation on the concentration of Vitamin D and its metabolites. The analysis of mouse hair samples revealed that only 25OHD_3_ was found among the tested forms of vitamin D when samples were subjected to the above-mentioned validated procedure. This finding likely reflects the higher physiological stability and abundance of 25OHD_3_ compared to other metabolites. The incorporation of metabolites into hair may also vary based on solubility and deposition efficiency, with 25OHD_3_ being preferentially accumulated. The levels of 25OHD_3_ varied among the different groups of mice (SDL, SDD, and DDD) and over the 6-month and 12-month periods. Specifically, at the 6-month mark, the average 25OHD_3_ levels were 17.7 ng/mg for SDL (N=15), 16 ng/mg for SDD (N=15), and below limit of quantification (BLQ) for DDD population of mice (N=10). Similarly, at the 12-month mark, the average 25OHD_3_ levels increased to 18.3 ng/mg for SDL, 17.7 ng/mg for SDD, and remained undetectable for DDD.

The results indicated a statistically significant difference in 25OHD_3_ levels between the SDL and DDD groups at both time points (p < 0.05). However, no significant difference was observed between the SDL and SDD groups, suggesting that light exposure plays a crucial role in maintaining higher 25OHD_3_ levels when dietary vitamin D is sufficient. Cohen’s d for differences between the SDL and DDD groups was calculated as 2.1 at 6 months and 2.3 at 12 months, indicating large effect sizes. Partial eta-squared values from the one-way ANOVA tests were 0.68 and 0.71 at the 6-month and 12-month intervals, respectively, reinforcing the strong statistical significance of these differences. *Post-hoc* analysis using Tukey’s HSD test further confirmed that the DDD group had significantly lower 25OHD_3_ levels compared to both the SDL and SDD groups at each time point (p < 0.01). Additionally, a paired t-test was conducted to evaluate the increase in 25OHD_3_ levels within each group over time, revealing a significant increase from 6 months to 12 months for both the SDL and SDD groups (p < 0.05), whereas the DDD group remained below the limit of quantification throughout the study period.

**Figure 3:**
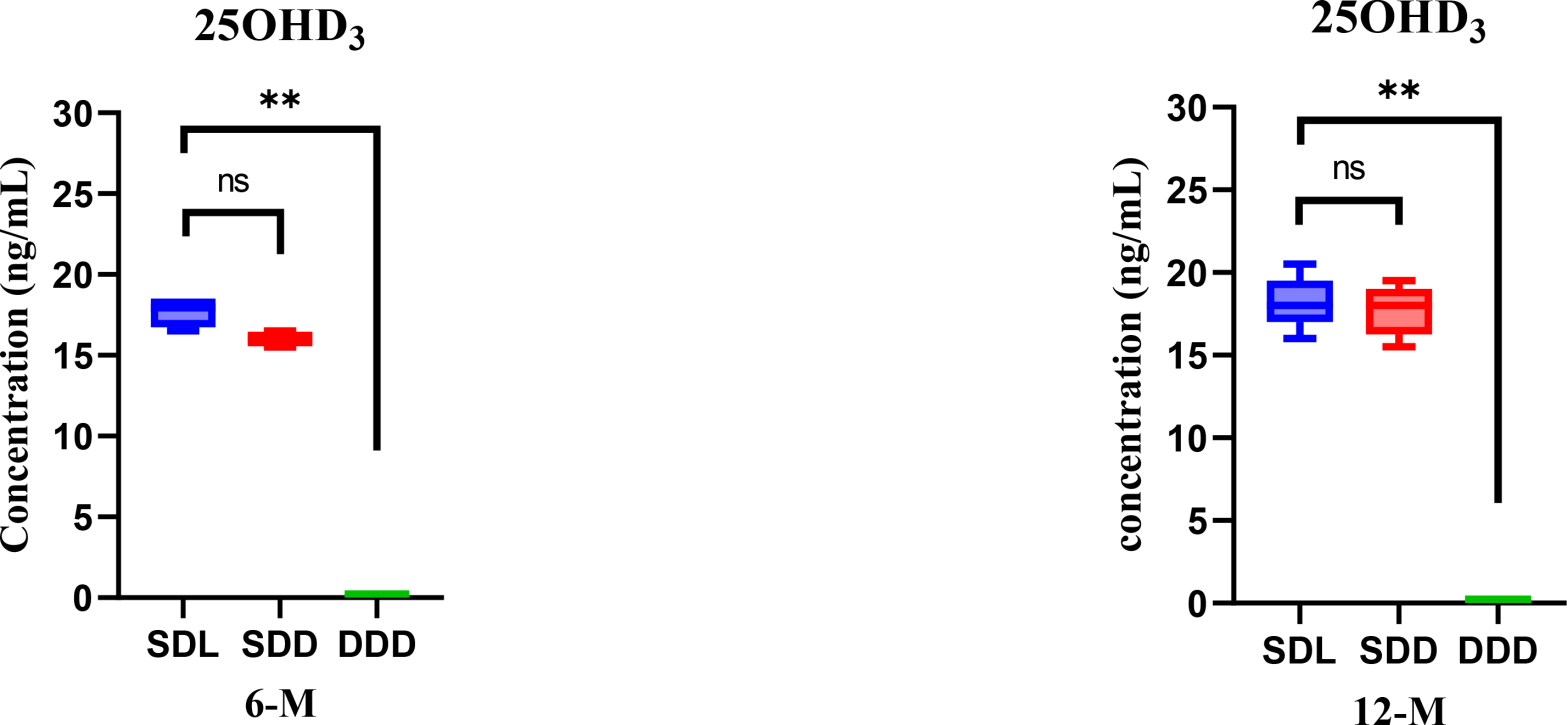
Comparison of 25OHD_3_ levels among SDL (N=15), SDD (N=15), and DDD (N=10) groups of mice hair at 6^th^ and 12^th^ month interval. Asterisk (**) represent significant differences between SDL and DDD groups. The error bars represent standard deviations. The differences between the results were considered significant if p < 0.05, refuting the null hypothesis (p < 0.05). “ns” means no significant difference.

The findings suggest that the concentrations of 25OHD_3_ in mouse hair samples vary based on dietary and light exposure conditions, with the SDL group of mice model consistently exhibiting the highest levels over the duration of the study. **Figure 3** illustrates the observed variations in 25OHD_3_ concentrations among the SDL, SDD, and DDD groups at both the 6-month and 12-month time points. The detection of 25OHD_3_ in hair highlights its potential as a long-term biomarker for vitamin D exposure, integrating both dietary and environmental sources. The significant differences between the SDL and DDD groups underscore the critical role of light exposure in maintaining adequate vitamin D levels, even when dietary sources are absent. The lack of significant differences between SDL and SDD groups suggests that dietary intake alone may be sufficient to maintain similar 25OHD_3_ levels as light exposure under certain conditions. These results support the notion that hair-based vitamin D analysis can serve as a complementary tool to serum measurements, especially in studies of chronic exposure or deficiency. These findings also highlight the role of light exposure in sustaining higher vitamin D levels, even when dietary intake is sufficient, and emphasize the biological significance of these results. While the assay’s capability to quantify up to eight forms of vitamin D is promising, further advancements in instrument sensitivity and sample pre-concentration techniques could potentially expand the scope of analytes quantified. The hair-based assay, focusing primarily on 25OHD_3_, presents a cost-effective and non-invasive alternative for long-term monitoring of vitamin D levels, complementing routine clinical tests and overcoming logistical challenges associated with traditional blood-based assays.

### 3.3 Serum sample analysis

**Figure 4:**
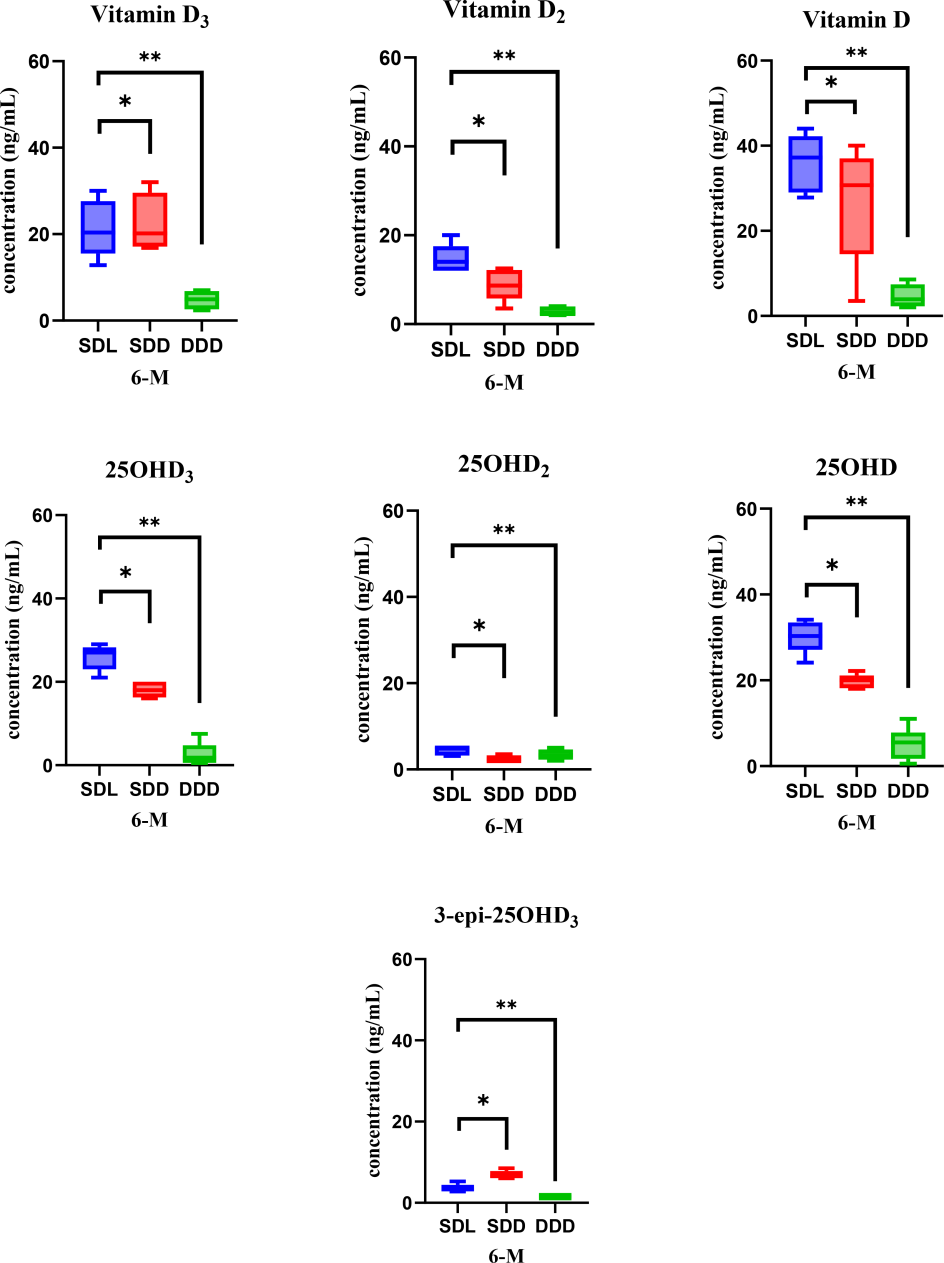
Representation of vitamin D metabolite’s concentrations in mouse serum of three study groups (SDL (N=15), SDD (N=15), and DDD (N=10)) at the 6-month mark. Asterisk (* & **) represent significant differences between SDL, SDD and DDD groups. The error bars represent standard deviations. The differences between the results were considered significant if p < 0.05, refuting the null hypothesis (p < 0.05). Vitamin D: 20–50 ng/mL (normal), <20 ng/mL (deficient). 25OHD: 15–40 ng/mL (normal), <15 ng/mL (deficient).

The validation results for analysis of Vitamin D and its metabolites in serum of mice has been already reported in our previous study (29). In this study, we aimed to further explain the variations in the concentration of vitamin D and its metabolites over the course of a year. Serum samples were collected twice annually, at the 6-month and 12-month marks, enabling a thorough analysis of changes in vitamin D levels. This analysis was conducted under consistent experimental and growth conditions for mice model throughout the year. At the 6-month analysis period, the concentrations of vitamin D and its metabolites were determined in the serum samples collected from the three study groups of mice: SDL (N=15), SDD (N=15), DDD (N=10) as shown in **Figure 4**. Statistical analysis (ANOVA) indicated significant differences between the SDL and DDD groups for both vitamin D and 25OHD levels (p < 0.01). Additionally, a significant difference was observed between the SDL and SDD groups for 25OHD concentrations (p < 0.05).

The analysis revealed notable variations in the concentrations of the metabolites among the groups. Specifically, the SDL group exhibited the highest average concentration of vitamin D (35.9 ng/ml), followed by the SDD group (26.8 ng/ml), and notably lower levels were observed in the DDD group (4.8 ng/ml). Similarly, for the 25OHD metabolite of vitamin D, the SDL group displayed the highest average concentration (30.3 ng/ml), followed by the SDD group (19.7 ng/ml), with the DDD group showing the lowest levels (5 ng/ml). The bar and whisker plots in **Figure 4** illustrate the concentrations of all analyzed vitamin D metabolites in the serum samples of mice at the 6-month mark. The error bars represent the standard deviation, indicating the variability in the concentrations within each group. As depicted in the **Figure 4**, the SDL group consistently exhibited higher concentrations of vitamin D and its metabolites compared to the other groups, while the DDD group consistently showed the lowest concentrations across all metabolites.

**Figure 5:**
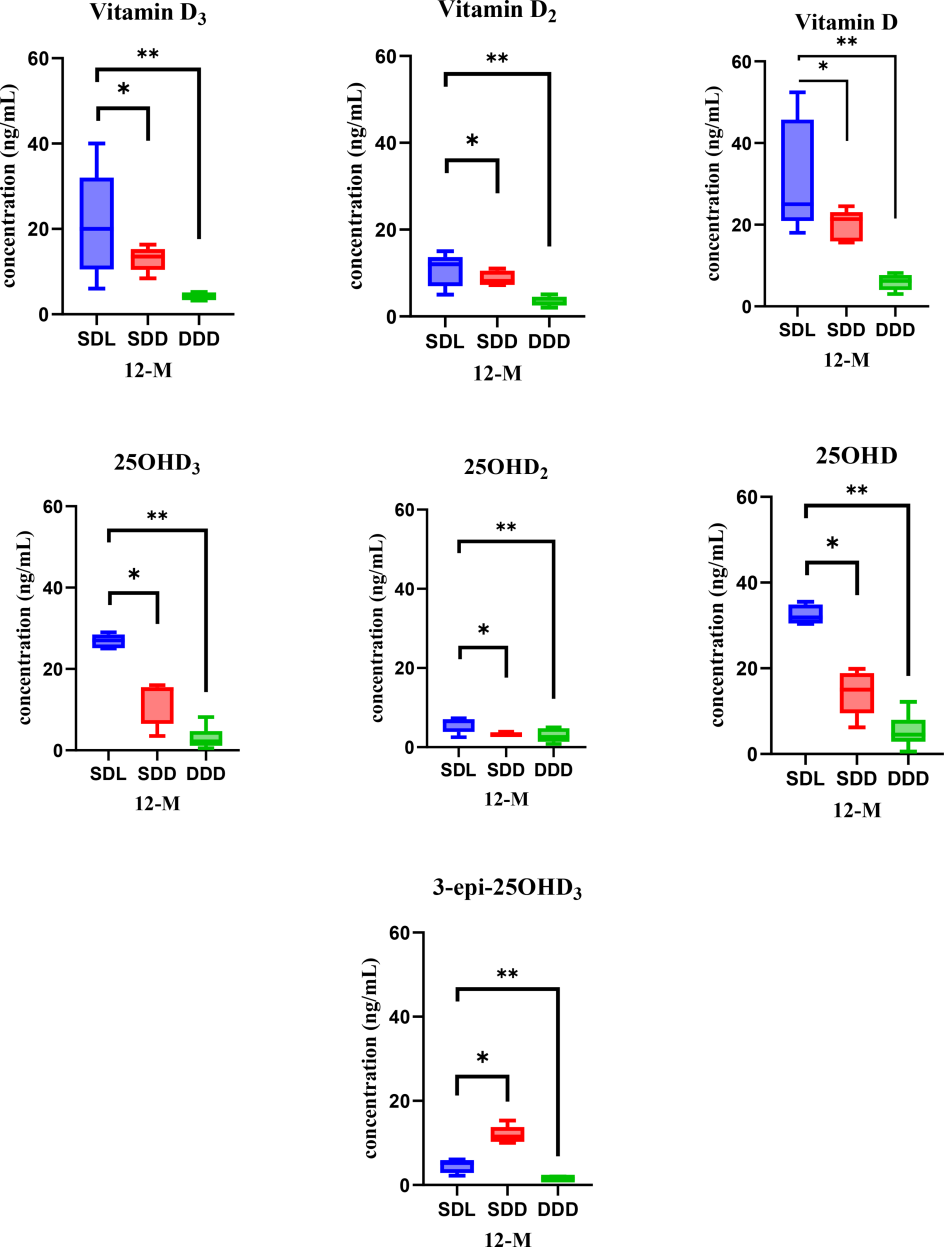
Bar and whisker plots showcasing vitamin D metabolite’s concentrations in mice serum of three study groups (SDL (N=15), SDD (N=15), and DDD (N=10)) at the 12-month mark. Asterisk (* & **) represent significant differences between SDL, SDD and DDD groups. The error bars represent standard deviations. The differences between the results were considered significant if p < 0.05, refuting the null hypothesis (p < 0.05). Vitamin D: 20–50 ng/mL (normal), <20 ng/mL (deficient). 25OHD: 15–40 ng/mL (normal), <15 ng/mL (deficient).

When analyzing the serum samples at the 12^th^ month of the study year, the concentration of 25OHD exhibited the highest levels across all samples, followed by vitamin D and 25OHD_3_. **Figure 5** presents the bar and whisker plots of all analyzed metabolites in mice serum at the 12^th^ month analysis, with error bars indicating the standard deviation. ANOVA results at this time point also indicated significant differences between SDL and DDD groups for both vitamin D and 25OHD (p < 0.01), while SDL showed higher levels than SDD for 25OHD (p < 0.05). The data reveal distinct concentration levels for each metabolite across the three experimental groups. Specifically, the SDL group demonstrates the highest concentrations for most metabolites, reflecting the influence of standard vitamin D intake and UV light exposure. Conversely, the DDD group exhibits significantly lower concentrations, indicating the impact of a vitamin D-deficient diet and absence of light exposure. These findings underscore the critical role of dietary vitamin D intake and light exposure in modulating vitamin D metabolite concentrations in the serum over time. Additionally, the variations observed among the experimental groups highlight the importance of environmental factors in shaping vitamin D metabolism in mice models.

Vitamin D metabolites, particularly 25OHD and its active form 1α25(OH)_2_D_3_, play critical roles in regulating endocrine functions. The observed variations in serum metabolite concentrations among the groups are reflective of the regulatory influence of vitamin D on calcium and phosphorus homeostasis, primarily mediated through the suppression of parathyroid hormone (PTH) secretion. The high concentrations of 25OHD observed in the SDL group may enhance PTH suppression, thereby maintaining calcium-phosphorus balance more effectively than the SDD or DDD groups. Conversely, the DDD group’s low serum concentrations likely reflect a deficiency-driven feedback loop, with reduced 25OHD levels failing to adequately regulate PTH and downstream metabolic pathways. These findings highlight the interplay between vitamin D status and systemic endocrine adaptation, emphasizing the importance of both dietary intake and light exposure in maintaining endocrine health (22, 25, 36, 68).

### 3.4 Statistical analysis

A comprehensive statistical analysis was conducted to evaluate differences in vitamin D and its metabolites among the three groups (SDL, SDD, and DDD) and across two time points (6 months and 12 months). The results indicated statistically significant differences in the concentrations of vitamin D and its metabolites between the SDL and DDD groups at both time points (p < 0.01). Significant differences were also observed between the SDL and SDD groups, particularly for 25OHD levels (p < 0.05), underscoring the role of UV light exposure in enhancing vitamin D metabolite levels. *Post-hoc* analysis confirmed that the DDD group consistently had significantly lower concentrations of vitamin D and its metabolites compared to both the SDL and SDD groups (p < 0.01).

To ensure sufficient statistical power, a *post-hoc* power analysis was performed based on the observed effect sizes for vitamin D metabolite concentrations across groups and time points. This analysis confirmed that the sample sizes (SDL: N=15, SDD: N=15, DDD: N=10) provided >80% power (α = 0.05) to detect significant differences. Effect sizes were calculated using Cohen’s d for pairwise comparisons and partial eta squared (η²) for ANOVA results to quantify the magnitude of observed differences. Paired t-tests were conducted to analyze temporal changes in metabolite levels within each group from 6 months to 12 months. The results revealed significant increases in both vitamin D and 25OHD levels in the SDL and SDD groups (p < 0.05), while the DDD group showed no significant changes, reflecting a persistent deficiency due to the absence of dietary supplementation and light exposure.

### 3.5 Correlation analysis between hair and serum samples

To evaluate the consistency of vitamin D levels between hair and serum, a correlation analysis was conducted on the concentrations of 25OHD_3_ in both matrices across the SDL, SDD, and DDD groups at 6 and 12 months. Our findings demonstrated a moderate positive correlation (r = 0.56, p < 0.05) between hair and serum 25OHD_3_ concentrations, suggesting that hair vitamin D levels reliably reflect serum levels over time. This correlation was most pronounced in the SDL group, which received UV light exposure and a standard vitamin D diet. Notably, the DDD group, which was vitamin D-deficient, consistently exhibited the lowest 25OHD_3_ levels in both hair and serum, reinforcing the reliability of hair samples for identifying deficiencies. The moderate correlation indicates that while hair captures long-term vitamin D status, serum levels may respond more dynamically to short-term dietary and environmental variations. This consistency between hair and serum vitamin D measurements supports the validity of hair as a viable biomarker for vitamin D status, particularly in studies aiming to monitor long-term exposure.

## 4 Discussion

The analysis of serum samples revealed substantial differences in the concentrations of vitamin D and its metabolites among the various experimental groups. This comparison of vitamin D metabolite concentrations allowed us to discern the impact of experimental conditions on serum vitamin D dynamics over time. The graphical representation in the **Figures 4**, **5** illustrates the levels of vitamin D metabolites in mouse serum after 6 months (6-M) and 12 months (12-M) period provides insights into these variations. These plots give a visual comparison of concentrations among different experimental groups. Each bar represents the concentration of a specific vitamin D metabolite, with error bars indicating the standard deviation. Significant differences observed between the SDL, SDD and DDD groups of mice across various parameters, including vitamin D and its metabolites. 1α25(OH)_2_D_3_, 1α25(OH)_2_D_2_ and 1α25(OH)_2_D were below limit of quantification (BLQ) and is not detected in the samples. These variations are attributed to differences in dietary intake and exposure to light, both of which significantly influence the levels of vitamin D metabolites in mice serum.

Comparing the SDL and DDD groups, it was evident that both diet and light exerted considerable effects on the concentrations of vitamin D metabolites in the blood. This observation underscores the importance of environmental factors in modulating vitamin D metabolism and maintaining optimal levels of its metabolites in serum. In particular, a significant difference in the concentrations of 25OHD_3_ metabolite was observed between the SDL and SDD groups, attributed to the influence of UV light. These findings suggest that UV light exposure enhances the production of vitamin D epimers in serum, either by stimulating their synthesis or by promoting their metabolic efficiency. The notable increase in Vitamin D concentrations in the SDL group, compared to the DDD and SDD groups, further supports the beneficial effects of a standard vitamin D intake coupled with UV light exposure on the levels of vitamin D metabolites. Furthermore, levels of vitamin D metabolites in serum were markedly low in the DDD group, indicative of a vitamin D-deficient diet and lack of light exposure. Conversely, both SDD and SDL groups exhibited substantially higher concentrations of these metabolites, underscoring the synergistic effect of dietary vitamin D intake and UV light exposure on maintaining optimal serum levels.

**Figure 6:**
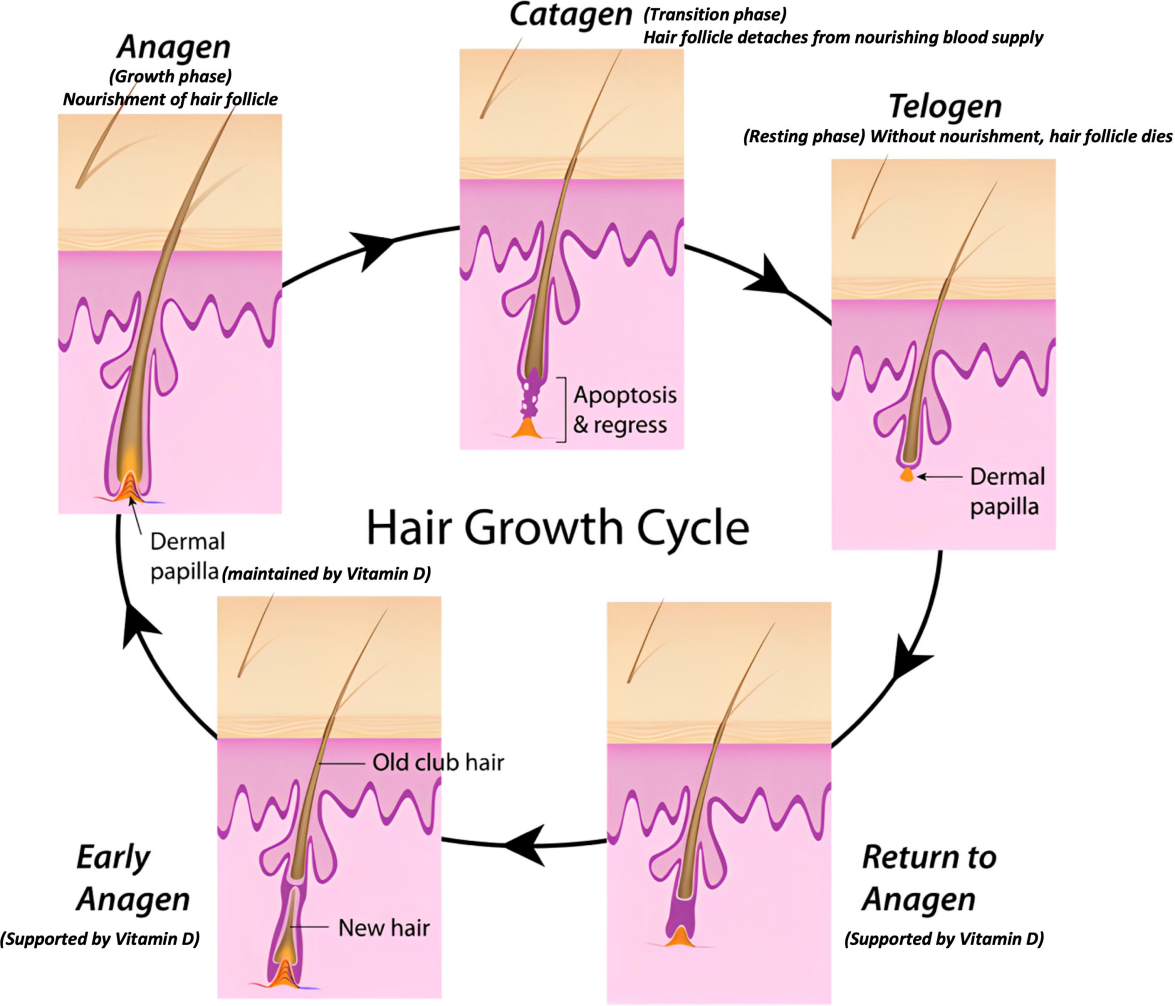
Schematic representation of the hair growth cycle and the role of vitamin D.

Hair grows in cycles comprising anagen (active growth), catagen (transition), and telogen (resting) phases, which influence metabolite incorporation into the hair shaft (**Figure 6**). During the anagen phase, systemic metabolites are actively deposited into growing hair, whereas metabolite deposition decreases significantly during the catagen and telogen phases as the follicle becomes metabolically inactive. Consequently, variability in the proportion of hair follicles in different phases at the time of collection can affect the metabolite levels observed (58, 69, 70). In this study, to mitigate the impact of hair growth cycle variability, we coordinated the growth cycle by shaving hair at the study’s onset and collected samples after six months of regrowth for the first time point and at 12 months for the second. This approach ensured that hair samples represented cumulative vitamin D metabolite deposition during stable growth phases, minimizing the influence of transient growth cycle variability. Hair composition, growth characteristics, and environmental exposure vary across different body regions, potentially affecting metabolite deposition and measurement. For example, dorsal hair is less exposed to contaminants such as grooming residues compared to ventral or tail hair and typically exhibits more uniform growth patterns. Additionally, differences in lipid content and structural properties across regions can alter the incorporation and retention of metabolites (70–72). This standardization minimized variability and enhanced reproducibility across experimental groups.0

The interpretation of these findings is further contextualized by the characteristics of the C57BL/6J mouse strain used in this study. This strain is widely recognized for its genetic predisposition to lower bone density, attributed to reduced bone formation rates and altered bone turnover dynamics. Histomorphometric studies have demonstrated that C57BL/6J mice exhibit impaired bone formation, characterized by reduced trabecular thickness and bone volume. These skeletal characteristics provide a valuable model for understanding genetic influences on vitamin D metabolism and bone health. The observed differences in vitamin D metabolite concentrations in the SDL and DDD groups align with the strain’s phenotype, emphasizing the critical role of dietary vitamin D intake and light exposure in mitigating the effects of lower bone density in this genetic model (60, 61).

The application of LC-MS/MS in this study highlights its utility as a powerful tool for exploring nutrigenomic aspects of hair biology. By precisely quantifying vitamin D metabolites in serum and hair samples, this methodology enables the investigation of how systemic and local vitamin D metabolism influences genetic and epigenetic regulation in hair follicles (57, 73, 74). Vitamin D status, for instance, could affect the expression of genes involved in keratinocyte differentiation, stem cell activation, and immune modulation, all of which are crucial for maintaining hair follicle health and promoting the hair growth cycle (5, 73, 75). Future applications of this technique could include correlating metabolite profiles with gene expression data to uncover molecular pathways through which vitamin D exerts its effects on hair follicle biology. The observed differences in vitamin D metabolite profiles among experimental groups provide a foundation for exploring their potential roles in gene regulation. Vitamin D metabolites, particularly 1α25(OH)_2_D, interact with the vitamin D receptor (VDR) to modulate the transcription of target genes. This interaction has been implicated in processes such as keratinocyte differentiation, immune response, and oxidative stress management within the hair follicle microenvironment. Elevated levels of 1α25(OH)_2_D in groups exposed to standard vitamin D diets and regular light cycles suggest enhanced VDR activity, which may positively influence gene expression patterns critical for anagen initiation and maintenance. These mechanisms provide a molecular framework linking vitamin D metabolism to nutrient-gene interactions in hair follicle development.

Vitamin D plays a pivotal role in regulating gene expression in hair follicles by binding to VDR, which subsequently interacts with vitamin D response elements (VDREs) in target genes. This interaction influences the transcriptional regulation of keratinocyte differentiation, essential for hair follicle integrity and normal hair growth cycle progression. Specifically, vitamin D metabolites may modulate the expression of genes regulating cell proliferation, differentiation, and apoptosis within the hair follicle matrix. These findings align with previous studies suggesting that vitamin D deficiency disrupts hair follicle cycling, potentially contributing to alopecia. The LC-MS/MS results presented here provide a platform for further exploration of these transcriptional dynamics under varying vitamin D statuses. The interplay between nutrient availability and gene expression in the hair follicle is a critical aspect of hair biology. Vitamin D metabolites are known to influence nutrient-gene interactions, particularly in regulating genes associated with the anagen, catagen, and telogen phases of the hair growth cycle. For instance, alterations in vitamin D levels can modulate β-catenin signaling pathways, which are crucial for the activation of hair follicle stem cells during anagen. Additionally, nutrient deficiencies may exacerbate telogen effluvium, a condition characterized by premature hair follicle dormancy. These insights underscore the significance of maintaining optimal vitamin D levels to support gene regulatory networks that govern hair growth and cycling. Our findings demonstrate that supplementation with vitamin D or exposure to UV radiation leads to a significant increase in circulating vitamin D metabolite concentrations. However, the mechanisms driving vitamin D epimerization and its differential regulation in cellular contexts remain poorly understood, warranting further investigation. It is essential to acknowledge the inherent physiological differences between mice and humans in vitamin D metabolism. While our results provide valuable insights into dietary intake and light exposure effects in mice, extrapolating these findings to humans requires cautious interpretation and further study. Human metabolism involves a combination of sunlight exposure and dietary intake for vitamin D synthesis, unlike the nocturnal dietary intake predominant in mice. These distinctions should inform future research to clarify the broader implications of these findings for human health (37, 58, 76–78).

Emerging evidence suggests that vitamin D status can influence epigenetic modifications, with profound implications for hair follicle biology. Vitamin D metabolites, particularly 1α25(OH)_2_D, have been shown to modulate DNA methylation patterns and histone modifications in keratinocytes. These epigenetic changes regulate the expression of genes critical for the hair growth cycle, such as those involved in stem cell activation, apoptosis, and immune response. Alterations in vitamin D-mediated epigenetic regulation may also contribute to conditions such as alopecia areata, characterized by dysregulation of immune-related genes. While our study did not directly assess epigenetic modifications, the findings provide a biochemical basis for future research into how vitamin D metabolites shape the epigenetic landscape of hair follicles. Incorporating analyses of methylation and histone modifications in future studies could yield valuable insights into the mechanisms linking vitamin D metabolism to nutrient-gene interactions in hair follicle development. The findings of this study have significant implications for understanding nutrient-gene interactions in hair follicle development. Vitamin D metabolites not only serve as markers of systemic nutrient status but also act as key regulators of hair follicle function through transcriptional and epigenetic mechanisms. By elucidating the relationships between metabolite profiles, gene expression, and hair follicle biology, this research contributes to a growing body of evidence supporting vitamin D’s role in maintaining hair health. Integrating LC-MS/MS data with transcriptomic and epigenomic analyses in future studies could provide deeper insights into how nutrient availability modulates gene regulatory networks in hair follicle biology. This integrative approach will enhance our understanding of the molecular underpinnings of hair growth and its response to environmental and dietary factors (74, 79, 80).

## 5 Conclusions

In this study, we successfully developed and validated a sensitive LC-MS/MS assay for the analysis of vitamin D and its metabolites in both mouse hair and serum samples. Unlike previous assays, our method is simple, precise, accurate, and it offers sensitivity, and improved precision, without the need for extensive sample pre-treatment and purification. Through the analysis of hair and serum samples collected over a year, we investigated the variations in vitamin D metabolite concentrations under different experimental conditions. Our results indicate that administering a standard vitamin D diet increased the levels of vitamin D metabolites in both mouse hair and serum. Furthermore, when combined with UV irradiation, the levels of metabolites were further elevated, suggesting a synergistic effect between diet and light exposure. These findings have important endocrine implications, highlighting the role of dietary intake and light exposure in maintaining vitamin D homeostasis. The validated assay offers potential for studying vitamin D-related endocrine disorders and monitoring treatment responses. Its application to non-invasive hair analysis provides a novel tool for long-term monitoring of vitamin D status, with promising utility in both research and clinical settings.

### 5.1 Future perspectives

This study highlights the potential of serum and hair-based assays for assessing vitamin D status, offering a non-invasive, sensitive approach to monitor vitamin D metabolism. Future efforts should focus on expanding the range of analytes to include additional vitamin D metabolites and refining the assay to become the gold standard for hair analysis. An important direction for future research would be to integrate analyses of key endocrine regulators, including parathyroid hormone (PTH) and fibroblast growth factor 23 (FGF23), to further elucidate the interplay between vitamin D metabolism and calcium-phosphorus homeostasis. These parameters could provide deeper mechanistic insights into the vitamin D endocrine axis and its role in systemic nutrient signaling. Additionally, exploring the expression of enzymes such as CYP27B1 and CYP24A1 in tissue samples would shed light on how vitamin D metabolites are processed in specific physiological contexts and under varying environmental conditions. Our findings also open the door to investigating the potential molecular and nutrigenomic implications of vitamin D metabolism. Future studies could leverage this assay to study how vitamin D as a micronutrient regulates gene expression in hair follicles and other tissues. Specifically, understanding the interactions between vitamin D status and nutrient-gene interactions affecting hair growth cycles, transcriptional regulation, and epigenetic modifications could provide valuable insights into vitamin D’s broader physiological roles. Translational research could further explore the application of this methodology to monitor treatment responses in vitamin D-related endocrine disorders such as rickets, osteoporosis, and chronic kidney disease. These applications would contribute to a more comprehensive understanding of how vitamin D metabolism influences disease progression and management. Investigating additional factors such as age, ethnicity, gender, and seasonal variations on vitamin D levels would further enrich our knowledge of vitamin D biology and its implications for diverse populations.

## Data Availability

The original contributions presented in the study are included in the article/supplementary material. Further inquiries can be directed to the corresponding author.
